# Are estrogen-related drugs new alternatives for the management of osteoarthritis?

**DOI:** 10.1186/s13075-016-1045-7

**Published:** 2016-06-28

**Authors:** Ya-Ping Xiao, Fa-Ming Tian, Mu-Wei Dai, Wen-Ya Wang, Li-Tao Shao, Liu Zhang

**Affiliations:** Department of Orthopedic Surgery, The Affiliated Hospital of North China University of Science and Technology, No. 73 Jianshe South Road, Tangshan, Hebei Province China; Medical Research Center, North China University of Science and Technology, Tangshan, China; Department of Orthopedic Surgery, Hebei Medical University, Shijiazhuang, China; Department of Pathology, School of Basic Medical Sciences, North China University of Science and Technology, Tangshan, China

**Keywords:** Osteoarthritis, Estrogen, Selective estrogen receptor modulators, Joint, Bazedoxifene

## Abstract

Osteoarthritis (OA) is a chronic degenerative disease involving multiple physiopathological mechanisms. The increased prevalence of OA after menopause and the presence of estrogen receptors in joint tissues suggest that estrogen could help prevent development of OA. This review summarizes OA research with a focus on the effects of estrogen and selective estrogen receptor modulators (SERMs). Preclinical studies and clinical trials of estrogen therapy have reported inconsistent results. However, almost all studies assessing SERM treatment have obtained more consistent and favorable effects in OA with a relatively safety and tolerability profiles. At present, some SERMs including raloxifene and bazedoxifene have been approved for the treatment of osteoporosis. In summary, estrogen-related agents may exert both a direct effect on subchondral bone and direct and/or indirect effects upon the surrounding tissues, including the articular cartilage, synovium, and muscle, to name a few. Estrogen and SERMs may be particularly favorable for postmenopausal patients with early-stage OA or osteoporotic OA, a phenotype defined by reduced bone mineral density related to high remodeling in subchondral bone. At present, no single drug exists that can prevent OA progression. Although estrogen-related drugs provide insight into the continued work in the field of OA drug administration, further research is required before SERMs can become therapeutic alternatives for OA treatment.

## Background

Osteoarthritis (OA) is a chronic, progressive disease that affects the entire joint organ and eventually leads to joint organ dysfunction [1]. An OA subset of high remodeling and/or low subchondral bone mineral density (BMD) may benefit from management with anti-resorptive agents to inhibit OA progression [2–5]. OA is the main cause of disability in the older population and is a socioeconomic burden worldwide.

Observational studies indicate that the prevalence of OA is increased immensely in postmenopausal women [6]. Further research has identified the presence of estrogen receptors (ERs) in joint tissues [7]. Moreover, the aromatase gene involved in estrogen secretion and ER gene mutation are associated with OA severity of the lower limb large joint [8]. Similarly, polymorphisms in the ERα gene might also be associated with a higher OA risk [9, 10]. Taken together, evidence strongly suggests that estrogen may be involved in the development of OA.

Selective estrogen receptor modulators (SERMs) are synthetic nonsteroidal agents with different chemical structures that elicit diverse estrogen agonist and antagonist activities within different tissues [11]. However, SERMs have shown consistent agonist activities in joint tissues. An ideal SERM would exert favorable tissue-selective estrogenic agonist activities in the bone, cardiovascular system, brain, urogenital system, vagina, and skin, with ER neutral or anti-estrogenic activities in the endometrium, breast, and pelvic floor [12, 13]. Importantly, SERM treatment has not resulted in any long-term estrogen treatment-related adverse events to date.

Recent studies have supported that estrogen or SERMs may have beneficial effects on joint tissues (Table 1). In this review, relevant English-language articles concerning the effects of estrogen or SERMs in OA progression or on joint tissues were identified using the PubMed database. The aim of this literature review was to identify evidence suggesting that early-stage OA patients, particularly osteoporotic OA patients, may benefit from treatment with estrogen or SERMs. The findings highlight that, at present, no single drug can prevent OA progression, while estrogen-related drugs analyzed together provide insight into the ongoing work on OA administration.

**Table 1:** Effects of estrogen-related drugs on joint tissues

Tissue	Main effects
Articular cartilage	Reduction of articular cartilage turnover and destruction, regulation of cartilage metabolism, improvement of mechanical properties
Subchondral bone	Regulation of bone growth and remodeling, promotion of matrix production and mineralization, regulation of osteoblast and osteoclast development and function
Synovial membrane	Decrease of the proliferation of rheumatoid arthritis-like synovial cells, decrease of proinflammatory cytokine production, reversion of experimental arthritis
Muscle	Promotion of myoblast proliferation and differentiation, reduction of muscle cell apoptosis, reversion of muscle atrophy and contractile dysfunction

## Estrogen therapy: inconclusive results

Direct binding of estrogen to ERs acts on joint tissues, protecting their biomechanical structure and function, thus maintaining overall joint health (Table 2). However, the exact effect of estrogen on OA remains controversial and in some cases inconsistent, likely owing to the methodological drawbacks or the varying OA phenotypes as detailed in the research.

**Table 2:** Effects of estrogen on joint tissues

Drug name	Type of study	Effects on joint tissues	Reference
Estradiol	In-vivo OVX + OA rabbits	Cartilage degeneration	[16]
β-estradiol	In-vivo OVX rabbits	Loss of glycosaminoglycans and collagen	[17]
17β-estradiol	In-vivo OVX rats	Decrease of CTX-II; prevention of cartilage lesions	[20]
17β-estradiol	In-vivo postmenopausal OA women	Decrease of 17β-estradiol after menopause	[21]
Estrogen	In-vivo postmenopausal OA women	Estrogen deficiency may lead to increase of serum IL-6	[22]
17β-estradiol	In-vivo murine with knee OA	Inhibition of tibial and patellar subchondral cortical thinning and tibial cartilage damage	[23]
17β-estradiol	In-vivo OVX + OA mice	Inhibition of bone resorption; decreased ADAMTS-4 and ADAMTS-5 expression	[19]
β-estradiol	In-vivo OA + OVX murine	Reduction of cartilage and bone turnover	[18]
β-estradiol	In-vivo OVX + ACLT murine	Regulation of intraarticular neurogenic inflammation	[24]
17β-estradiol	In-vivo OA + OVX murine	Potentiation of cartilage degradation and subchondral bone erosion and mRNA expression of Fas, FasL, caspase 3, and caspase 8	[25]
17β-estradiol	In-vitro rabbit chondrocytes	Upregulation of type II collagen gene	[26]
17β-estradiol	In-vitro cow mature joint cartilage	Prevention injury-related cell death and GAG release	[27]
17β-estradiol	In-vitro rabbit chondrocytes	Inhibition of doxorubicin-induced apoptosis	[28]
17β-estradiol	In-vitro rat OA chondrocytes	Promotion of chondrocyte proliferation	[29]
Oral estrogen	CSS osteoporotic white women	Reduction of risk of any hip OA	[31]
HRT	CSS women around menopause	Inverse association of current HRT use and radiological OA of the knee	[35]
Oral estrogen	CSS women with OA	No positive association of estrogen use with radiographic knee OA	[36]
ERT	CSS older women	Protection moderately against worsening of radiographic knee OA, but not statistically significant	[37]
ERT	CSS women	Nonsignificant protective effect for incident knee osteophytes	[38]
CEE	RCT community-dwelling women	Lower rates of any arthroplasty	[32]
Estrogen	CSS women	Lower subchondral bone attrition and bone marrow edema-like abnormalities	[33]
Estrogen	CSS, older women	No significant correlation with knee replacement of OA	[39]
HT	Prospective study, women around menopause	Correlation highly with the hip or knee replacement rates of OA	[40]
17β-estradiol	RCT postmenopausal OP women	Decrease of levels of COMP	[34]

*OA* osteoarthritis, *OP* osteoporosis, *OVX* ovariectomy, *CTX-II* C-terminal cross-linked telopeptide type II collagen, *HRT* hormone replacement therapy, *ERT* estrogen replacement therapy, *COMP* cartilage oligomeric matrix protein, *ACLT* anterior cruciate ligament transaction, *CEE* conjugated equine estrogens, *HT* hormonal therapies, *CSS* cross-sectional study, *RCT* randomized controlled trial

### Preclinical studies

A systematic review comprising controlled studies found estrogen to have confounding effects on articular cartilage in ovariectomized (OVX) animals [14]. Interestingly, only 11 out of 22 animal studies report beneficial actions of estrogen on OA, suggesting that the estrogenic effect is inconclusive, which is consistent with the majority of recently published literature [15]. In fact, intraarticular injection of estrogen was reported to actually damage the knee articular cartilage in OVX rabbits [16, 17], with long-term estrogen treatment being beneficial for articular cartilage in other animal models [18, 19]. Early studies of estrogen administration may therefore overestimate the positive effect of estrogen on articular cartilage [20].

In-vivo estrogen treatment has shown inconclusive results in OA. Gao et al. [21] found that serum-free estradiol levels and total serum estradiol levels were significantly lower in postmenopausal compared with premenopausal OA women. Afzal and Khanam [22] found that estrogen deficiency may lead to increased serum IL-6 in postmenopausal patients with OA, which has been found to promote OA progression. In a murine model of knee OA, exogenous estrogen suppressed tibia and patella subchondral cortical bone thinning and prevented patellar cartilage damage, supporting an etiological role for altered estrogen signaling in OA [23]. Similarly, in an osteopenic mouse, estrogen treatment recovered bone mass of the subchondral bone, but although it reduced the expression of cartilage ADAMTS-4 and ADAMTS-5, cartilage damage was not significantly prevented [19]. However, estrogen has been shown to improve the histological integrity of articular cartilage and reduced cartilage and bone turnover in a murine OA model [18]. Furthermore, in an OA OVX rat model, 17β-estradiol treatment significantly reduced the density of substance P and calcitonin gene-related peptide immunoreactive nerve fibers in the synovial membrane, suggesting that estrogen partly regulates intraarticular neurogenic inflammation in OA joints by modulating the expression of neuropeptides in the synovial membrane [24]. However, in an iodoacetate-induced temporomandibular joint OA rat model, estrogen administration promoted cartilage degeneration, subchondral bone erosion, and expression of apoptosis genes [25]. Intraarticular injection of estrogen was also reported to actually damage the knee articular cartilage in rabbits OA models [16, 17].

Estrogen appears to have a potential protective effect on chondrocytes in vitro. In rabbit articular chondrocytes, 17β-estradiol has been shown to upregulate collagen type II expression by Sp1/3, Sox-9, and ERα [26]. In another study, 17β-estradiol treatment prevented injury-related cell death and glycosaminoglycan release in mature articular cartilage explants, suggesting that 17β-estradiol may be useful for treating either cartilage-related sports injuries or OA [27]. Kumagai et al. [28] also found that 17β-estradiol suppressed doxorubicin-induced apoptosis by blocking volume-sensitive Cl^–^ current in rabbit articular chondrocytes. In cultured chondrocytes from a rat OA model, 17β-estradiol promoted chondrocyte proliferation via the PI3K/Akt pathway [29].

### Clinical studies

Consistent with these preclinical studies, comprehensive analysis of a multitude of clinical studies has shown that the effects of estrogen on hand OA or other joint OA failed to reach clear conclusions [15, 30].

On the one hand, estrogen appears to have a potential protective effect on OA in many clinical studies. A cross-sectional study found that postmenopausal women who received long-term estrogen treatment had a significantly reduced risk of any radiographic hip OA, indicating that postmenopausal estrogen management may ameliorate hip OA [31]. In the Women’s Health Initiative study [32], women receiving conjugated equine estrogens had significantly lower rates of arthroplasty, particularly hip replacement rates. In a cross-sectional study, knee MRI scans identified that women receiving estrogen had significantly less subchondral bone attrition and bone marrow edema-like abnormalities in the knee compared with nontreated women [33]. Likewise, a case–control study found that estrogen treatment significantly reduced the serum level of cartilage oligomeric matrix protein, a marker associated with cartilage degeneration, suggesting that estrogen treatment may be a novel treatment modality to prevent OA joint degeneration [34].

Conversely, estrogen seems to have no protective or even damaging effect on OA in some other clinical studies. Two of the largest observational studies, the Britain Chingford Study and the US Framingham Osteoarthritis Study, and subsequent follow-up of these two studies found that estrogen treatment did not significantly reduce the radiographic severity of knee OA or hand OA [35–38]. In a recent longitudinal observational study, estrogen therapy had no significant correlation with OA knee replacement [39]. Furthermore, estrogen treatment is reported to increase the joint replacement rate of OA, which conflicts with its suggested protective effect [15]. A prospective study showed that postmenopausal estrogen treatment increased the risk of hip or knee replacement of OA [40].

In summary, although considerable studies have found that estrogen may have potential protective effects on OA joints, the exact effect of estrogen on OA remains controversial. The inconclusive results of estrogen in OA may be due to the following reasons. First, estrogen may have different effects on the initiation and progression of OA. Increasing numbers of experimental and human studies have demonstrated the existence of remodeling abnormalities in the subchondral bone, with increased bone turnover and subsequent bone loss in early stages of OA [41, 42]. These changes are followed by reduced bone turnover and further subchondral sclerosis in the late stages of OA [41–43]. Consequently, estrogen could decrease bone turnover and may have potential beneficial effects for early-stage OA. Conversely, the prevention of bone loss may result in effects that are not beneficial or are even harmful for late-stage OA, with some studies showing an association between high bone density and radiographic OA changes [36, 38]. Second, estrogen may have a beneficial effect on only certain subtypes of OA. Depending on the ratio between formation and resorption, subchondral bone remodeling can culminate in either a sclerotic or an osteoporotic phenotype. Patients with osteoporotic OA may thus achieve clinical and structural benefit from estrogen intervention [2, 3, 5].

Finally, outcome measurements in these studies vary widely. Although some studies have reported moderate but nonsignificant protective effects [36–38], those detecting serologic or radiographic changes have shown an overall protective effect [31, 33–35]. Conversely, inconsistent results are apparent in studies using joint replacement patients [32, 40]. In addition to differences in trial design, the association between estrogen replacement therapy and the incidence of joint replacement may be affected by nonbiological factors. For example, women who take estrogen replacement therapy may have easier access to health services and, as such, may be more likely to have a joint replacement for existing OA. Furthermore, it is reported that women that have undergone knee replacement are in higher socioeconomic groups and are more likely to have other operations, such as hysterectomy [40].

Taken together, although estrogen treatment elicits a potential protective effect on OA, the identification of OA patient phenotypes and specific OA stages should be considered alongside therapeutic interventions in future studies, which may lead to clearer conclusions regarding estrogen therapy on OA progression.

## SERM treatment: consistent evidence

SERMs are a specific type of ER-binding estrogen that have selective effects on target tissues. Present studies support that SERMs have a more consistent beneficial effect on bone tissue, and therefore some have been approved for treatment of osteoporosis, such as lasofoxifene and bazedoxifene in Europe and the USA [44]. Subsequent studies have found that SERMs also have a beneficial role in other joint tissues, thus maintaining joint health as a whole. Compared with the controversial effects of estrogen administration on OA, SERMs have more stable and favorable effects on OA.

### Preclinical studies

Basic research has shown that SERMs inhibit destruction of articular cartilage and subchondral bone, delaying OA progression. Meanwhile, SERMs have an anti-inflammatory effect and prevent OA-like joint degeneration as a whole (Table 3).

**Table 3:** Effects of selective estrogen receptor modulators on joint tissues

Drug name	Type of study	Effects on joint tissues	Reference
Tamoxifen	In-vivo rabbit with OVX + MMX-OA	Reduction of cartilage damage	[48]
	In-vivo rabbit with MMX-OA	Reduction of cartilage damage	[46]
	In-vivo intact male rabbit with OA	Reduction of cartilage damage	[47]
	In-vivo rabbit with OVX + MMX-OA	Antagonism of chondrodestructive effects of high-dose estradiol intraarticular administration	[45]
CHPPPC	In-vivo Sprague–Dawley rats with OVX	Inhibition of the OVX-induced acceleration of bone and cartilage turnover, and suppression of cartilage damage	[49]
Levormeloxifene	In-vivo Sprague–Dawley rats with OVX	Prevention of the OVX-induced cartilage and bone changes	[50]
	RCT postmenopausal women	Decrease of CTX-I I by approximately 50 %	[50]
Lasofoxifene	In-vivo DBA/1 mice with OVX + arthritis	Reduction of the grade of histologic synovitis and erosions on cartilage and bone	[51]
Bazedoxifene	In-vivo DBA/1 mice with OVX + arthritis	Reduction of the grade of histologic synovitis and erosions on cartilage and bone	[51]
	In-vivo cynomolgus monkeys with OVX	Inhibition of OVX-induced vertebral deterioration of structural properties	[52]
	Prospective study in postmenopausal women with type 2 diabetes	Improvement of bone resorption markers	[57]
	RCT in postmenopausal women with OP	Lowered significantly the cumulative incidences of new vertebral fractures and maintained total hip bone mineral density	[58]
	Exploratory analysis women with increased fracture risk	Geometry-related improvements in bone strength	[59]
	RCT in women with menopausal symptoms	Improvement of lumbar spine and total hip BMD	[60]
	RCT in postmenopausal women with OP	Reduction of the incidences of vertebral and nonvertebral fractures	[61]
Raloxifene	In-vitro rat OA-like chondrocytes	Ceases or reduces the matrix degeneration in OA	[53]
	In-vitro human OA-like chondrocytes	Augmented in proteoglycans and a significant decrease of MMP-3 and NO levels	[54]
	Cross-sectional study in older women with knee OA	Less subchondral bone attrition and bone marrow edema-like abnormalities	[33]
	RCT in postmenopausal women with back or knee pain	Amelioration of bone and joint pain	[62]
Genistein	In-vitro human OA-like chondrocytes	Decrease of NO and IL-1β level in supernatant	[55]

*OA* osteoarthritis, *OVX* ovariectomy, *OP* osteoporosis, *MMX-OA* medial meniscectomy-induced OA, *CHPPPC cis*-3,4-7-hydroxy-3-phenyl-4-(4-(2-pyrrolidinoethox) phenyl)chromane, *RCT* randomized controlled trial, *CTX-II* C-terminal cross-linked telopeptide type II collagen, *BMD* bone mineral density, *MMP* matrix metalloproteinase, *NO* nitric oxide

Intraarticular administration of tamoxifen was shown to reduce cartilage damage and antagonize chondrodestructive effects of high-dose estradiol in different rabbit OA models [45–48]. Tamoxifen played a protective effect on articular cartilage in intact male rabbits, suggesting that its therapeutic effect might not only be associated with its anti-estrogenic role [47]. Furthermore, both levormeloxifene and *cis*-3,4-7-hydroxy-3-phenyl-4-(4-(2pyrrolidinoethoxy)phenyl)chromane suppressed OVX-induced acceleration of bone and cartilage turnover and ameliorated destruction of cartilage in female rats [49, 50]. Andersson et al. [51] found recently that the anti-osteoporotic drugs lasofoxifene and bazedoxifene ameliorated cartilage and bone lesions and the histologic grade of synovitis, indicating that they are potent inhibitors of joint inflammation and destruction of cartilage and bone in experimental arthritis. Similarly, Saito et al. [52] found that bazedoxifene inhibited OVX-induced deterioration of structural properties of vertebral cancellous bone in monkeys and improved bone strength.

The consistent effect of SERMs in vivo indicates a protective effect on chondrocytes. Kavas et al. [53] reported that 1 μM of raloxifene reduced expression of OA-related genes, apoptosis, and extracellular matrix-degrading enzymes, and increased extracellular matrix deposition and improved mechanical properties in rat articular chondrocytes with OA-like degeneration. However, these effects were reversed with increased dose, demonstrating that low-dose raloxifene has the potential to cease or decrease cartilage degeneration in OA. In cultured human chondrocytes, when raloxifene and IL-1β were coincubated in the culture medium, proteoglycans were significantly and dose-dependently augmented, and matrix metalloproteinase-3 and nitric oxide (NO) levels were significantly decreased, demonstrating that raloxifene antagonized IL-1β-induced OA-like chondrocyte changes [54]. In another study, the natural SERM genistein antagonized lipopolysaccharide-induced OA-like chondrocyte changes with a significant decrease of COX-2 protein and NO level in the supernatant of cultured human chondrocytes, indicating that genistein may maintain joint health through an anti-inflammatory effect [55].

### Clinical studies

SERMs appear to have protective effects on joint tissues. SERMs regulate metabolism of articular cartilage and subchondral bone, maintain their normal biomechanical structure and performance, and possibly delay disease progression of OA. In addition, SERMs may have a potential analgesic effect in OA. Moreover, SERMs may play protective roles on the synovium and other joint tissues, maintaining joint health as a whole.

In the Health, Aging and Body Composition Study cohort, raloxifene significantly reduced subchondral bone attrition, bone marrow edema-like abnormalities, and knee pain according to Western Ontario and McMaster Universities Arthritis Index scores when compared with the control group [33]. A further study found that SERMs have a positive effect on cartilage metabolism. In this placebo-controlled trial, levormeloxifene reduced the urinary excretion of C-terminal cross-linked telopeptide type II collagen (CTX-II) by approximately 50 % and restored CTX-II levels to the premenopausal range compared with the control group, indicating that levormeloxifene inhibits degeneration of articular cartilage [50]. In another study, raloxifene not only reduced the CTX-II level, but also decreased the level of the bone resorptive marker CTX-I in postmenopausal women [56]. Interestingly, when women ceased levormeloxifene treatment, CTX-II levels returned to baseline level, while CTX-I was still strongly suppressed. This suggests that SERMs may play a short-lived role on cartilage and a long-term role on bone [56]. In recent studies, bazedoxifene not only prevented bone loss and maintained BMD by reducing bone turnover in postmenopausal women, but also improved the microstructure of bone. Consequently, bazedoxifene enhanced bone strength and reduced the risk of vertebral and nonvertebral fractures in postmenopausal women [57–61]. Remarkably, Fujita et al. [62] reported that combined raloxifene and alfacalcidol treatment appeared to be more effective on bone and joint pain than alfacalcidol alone in postmenopausal women with either back or knee pain alone, or both, according to electroalgometry and visual rating scale measurements.

In summary, postmenopausal women with osteoporotic OA might benefit from treatment with SERMs. The mechanisms of action of SERMs within joint tissues are being gradually elucidated [53, 54, 63]. In addition to the role of SERMs on ERs and their interaction with coregulator proteins to produce transcriptional complexes, some clinical effects of SERMs may involve rapid actions mediated by G protein-coupled estrogen receptor 1 (GPER1), with subsequent activation of the PI3K/Akt and/or PKC/MAPK pathways. Consequently, tamoxifen and raloxifene have been identified as GPER1 agonists [53].

## Estrogen-related drug mechanisms of action in OA

Recent research has confirmed that significant changes in the subchondral bone occur during OA progression [64]. Key changes in the subchondral bone include high bone turnover with decreased BMD and bone biomechanical structural damage in the early stages of OA, which either coincide with or precede cartilage degeneration [64]. Subchondral bone degeneration may be the trigger for changes in the cartilage biomechanical and biochemical microenvironment, thus promoting cartilage erosion and ultimately OA progression [65]. Consequently, subchondral bone is a potential therapeutic target, and drugs acting on subchondral bone represent potential disease-modifying OA drugs [2–4]. Similarly, the main pathological change in OA is degeneration of the articular cartilage that promotes subchondral bone lesions during progression of OA, particularly in late OA stages when cartilage erosion is extensive [4, 43]. Therefore, subchondral bone and cartilage are strongly dependent on each other during the progression of OA. In short, OA disease-modifying drugs must be able to act on both of these joint tissues to prevent the development and progression of OA.

Estrogen-related drugs that act on both subchondral bone and cartilage are good candidates for early-stage OA treatment, especially osteoporotic OA. These drugs are potent in antagonizing bone resorption, which can effectively decrease bone remodeling and prevent subchondral bone loss and the deterioration of microarchitecture and biomechanical properties [18, 19, 23]. Thus, the protective effect of these drugs on articular cartilage may be an indirect effect through protection of the subchondral bone. Additionally, these drugs directly target cartilage tissue, preventing cartilage damage and maintaining healthy cartilage [26, 66]. In addition to the direct or indirect protective role of these drugs on articular cartilage, subchondral bone, and the surrounding joint tissues, including the synovium and muscle, the joint tissues themselves interact with each other, thus maintaining joint organ homeostasis as a whole and finally delaying joint degeneration [56].

Moreover, the beneficial effect of estrogen-related drugs on OA may be, at least in part, associated with amelioration of the abnormal mechanical stress via or by regulating ER. Mechanical stress has an important role in the pathogenesis of OA [67], whereby abnormal mechanical stress is reported to promote deterioration of the subchondral bone and articular cartilage during OA progression [68]. A recent study has reported that estrogen reduces mechanical injury-related cell death and proteoglycan degradation in an ER-mediated pathway in mature articular cartilage. This suggests that estrogen agents ameliorate abnormal mechanical stress to protect cartilage-related sports injuries or OA [27].

On the contrary, downregulation of ER expression is evident during cartilage degeneration [69, 70], which may be associated with abnormal mechanical stress [27]. More recently, lower serum estrogen has been shown to downregulate ER expression, with estrogen therapy upregulating ER expression [70, 71], a finding that may relate to the beneficial effects of estrogen agents on OA. In fact, these changes are similar to ER changes in disc degeneration and its estrogen therapy [69, 72].

In summary, abnormal mechanical stress changes the articular microenvironment to decrease expression of ERs, which is associated with subsequent joint degeneration. We therefore hypothesize that the upregulated expression of ER by estrogen may correlate with amelioration of abnormal mechanical stress, a hypothesis that warrants further investigation.

## Efficacy of estrogen-related drugs on joint tissues

Current observational studies suggest that estrogen may be involved in the progression of OA and have potential protective effects on joint tissues. However, some studies suggest that the role of estrogen is controversial and warrants further study. In contrast, preclinical and clinical studies indicate that SERMs not only have consistently positive effects on OA [33, 36, 38, 39, 53, 54], but also significantly reduce estrogen treatment-related adverse events. However, the positive estrogen-like effects of SERMs on bone tissue are weaker than those of estrogen [44], and therefore need to be strengthened. A number of reasons may account for this interesting phenomenon. First, estrogen has an extensive effect in vivo, regulating many metabolic pathways within various tissues [73, 74]. However, the interaction of these metabolic pathways may weaken the effect of estrogen on articular tissues in vivo. Conversely, SERMs exhibit tissue-specific ER antagonist or agonist activity, with selective effects on specific tissues [11–13]. Consequently, the impact of other metabolic pathways on the effect of SERMs in articular tissues is weaker than that of estrogen. SERMs could thus produce greater estrogen-like effects in vivo [50]. New estrogen-like drugs are continuously reported. For example, tissue-selective estrogen complexes (TSECs) are a combination of an estrogen and one SERM, and demonstrate many features of ideal SERMs [14]. Theoretically, estrogen could reinforce the positive role of SERMs on bone tissue, making the effects of TESCs on bone tissue more effective than SERMs, which has been verified in a clinical trial [75]. Conversely, the many adverse reactions of estrogen could be antagonized by SERMs. We speculate that the effects of TESCs on joint tissues may be greater than those of SERMs, with a generally desirable safety and tolerability profile. In short, TESCs may become candidates for OA drugs in the future.

## Safety and tolerability of estrogen-related drugs

Research has revealed that SERMs are suited for the treatment of OA with relatively favorable safety and tolerability profiles.

Long-term estrogen therapy stimulates breast and endometrial hyperplasia with significantly increased risk of breast and endometrial cancer [73]. Moreover, this drug increases the risk of cardiovascular events and stroke, especially thromboembolic diseases [74]. These adverse effects severely limit the clinical application of estrogen.

SERMs selectively act on ERs in target tissues and the adverse events of SERMs are significantly reduced compared with estrogen [11]. Almost all SERMs have anti-estrogenic action in the breast and do not increase the risk of breast cancer. Moreover, some SERMs such as tamoxifen, toremifene, and raloxifene can be used for the treatment or prevention of estrogen-sensitive breast cancer [44]. SERMs have relatively varied effects on the uterus. Furthermore, very few SERMs, such as tamoxifen, stimulate endometrial proliferation with the increased risk of endometrial cancer. In fact, the majority of SERMs have neutral or anti-estrogenic effects on the endometrium and do not increase the risk of endometrial cancer [13]. In addition, most SERMs do not increase the risk of cardiovascular events [11]. Although some SERMs are reported to slightly increase the incidence of hot flashes or vulvar vaginal atrophy, the symptoms are mild and do not affect the clinical application of SERMs. Currently, the primary limit of clinical application of most SERMs is the increased risk of venous thrombosis embolism [44].

## Conclusion

At present, the roles of estrogen in joint tissues or OA are controversial. However, SERMs have consistently protective effects on joint tissues or in OA with relatively favorable safety and tolerability profiles. SERMs and estrogen may represent therapeutic options to treat joint diseases in the future. In particular, SERMs and estrogen may be beneficial for postmenopausal women with osteoporotic OA or early-stage OA [2, 3, 5]. Although, there is a wide range of SERMs, their chemical structure and biological function are quite complex. Therefore, it is difficult to identify the optimal types of SERMs to treat OA. Further research is needed to identify the most suitable types of SERMs to treat OA and to clearly identify their action mechanisms on joint tissues. A new group of estrogen-related drugs, TSECs are reported to have a beneficial effect on bone tissue [75, 76]. Furthermore, TSECs may have the potential to protect other joint tissues, suggesting they may become favorable therapeutic alternatives for treatment of OA. These findings warrant further clarification in future preclinical and clinical studies.

## Abbreviations

ADAMTS, a disintegrin and metalloproteinase with thrombospondin motifs; Akt, protein kinase B; BMD, bone mineral density; COX-2, cycloxygenase-2; CTX-I, C-terminal cross-linked telopeptide type I collagen; CTX-II, C-terminal cross-linked telopeptide type II collagen; ER, estrogen receptor; GPER, G protein-coupled estrogen receptor 1; IL, interleukin; MAPK, mitogen-activated protein kinase; NO, nitric oxide; OA, osteoarthritis; OVX, ovariectomized; PI3K, phosphatidyl inositol 3-kinase; PKC, protein kinase C; SERM, selective estrogen receptor modulator; Sox-9, Sry related HMG box-9; TSEC, tissue selective estrogen complex

## References

[1] Malfait AM (2016). Osteoarthritis year in review 2015: biology. Osteoarthritis Cartilage..

[2] Roman-Blas JA, Herrero-Beaumont G (2014). Targeting subchondral bone in osteoporotic osteoarthritis. Arthritis Res Ther..

[3] Roman-Blas JA, Castaneda S, Largo R, Lems WF, Herrero-Beaumont G (2014). An OA phenotype may obtain major benefit from bone-acting agents. Semin Arthritis Rheum..

[4] Karsdal MA, Bay-Jensen AC, Lories RJ, Abramson S, Spector T, Pastoureau P (2014). The coupling of bone and cartilage turnover in osteoarthritis: opportunities for bone antiresorptives and anabolics as potential treatments?. Ann Rheum Dis..

[5] Herrero-Beaumont G, Roman-Blas JA (2013). Osteoarthritis: Osteoporotic OA: a reasonable target for bone-acting agents. Nat Rev Rheumatol..

[6] Srikanth VK, Fryer JL, Zhai G, Winzenberg TM, Hosmer D, Jones G (2005). A meta-analysis of sex differences prevalence, incidence and severity of osteoarthritis. Osteoarthritis Cartilage..

[7] Roman-Blas JA, Castaneda S, Largo R, Herrero-Beaumont G (2009). Osteoarthritis associated with estrogen deficiency. Arthritis Res Ther..

[8] Riancho JA, Garcia-Ibarbia C, Gravani A, Raine EV, Rodriguez-Fontenla C, Soto-Hermida A (2010). Common variations in estrogen-related genes are associated with severe large-joint osteoarthritis: a multicenter genetic and functional study. Osteoarthritis Cartilage..

[9] Yin YW, Sun QQ, Hu AM, Wang Q, Liu HL (2015). Association of rs9340799 polymorphism in estrogen receptor alpha gene with the risk of osteoarthritis: evidence based on 8,792 subjects. Mol Genet Genomics..

[10] Wang Q, Yan XB, Sun QQ, Hu AM, Liu HL, Yin YW (2015). Genetic polymorphism of the estrogen receptor alpha gene and susceptibility to osteoarthritis: evidence based on 15,022 subjects. Curr Med Res Opin..

[11] Pinkerton JV, Thomas S (2014). Use of SERMs for treatment in postmenopausal women. J Steroid Biochem Mol Biol..

[12] Taylor HS (2009). Designing the ideal selective estrogen receptor modulator—an achievable goal?. Menopause..

[13] Maximov PY, Lee TM, Jordan VC (2013). The discovery and development of selective estrogen receptor modulators (SERMs) for clinical practice. Curr Clin Pharmacol..

[14] Sniekers YH, Weinans H, Bierma-Zeinstra SM, van Leeuwen JP, van Osch GJ (2008). Animal models for osteoarthritis: the effect of ovariectomy and estrogen treatment—a systematic approach. Osteoarthritis Cartilage..

[15] de Klerk BM, Schiphof D, Groeneveld FP, Koes BW, van Osch GJ, van Meurs JB (2009). Limited evidence for a protective effect of unopposed oestrogen therapy for osteoarthritis of the hip: a systematic review. Rheumatology (Oxford).

[16] Tsai CL, Liu TK. Estradiol-induced knee osteoarthrosis in ovariectomized rabbits. Clin Orthop Relat Res. 1993;291:295–302. 8504610

[17] Hashem G, Zhang Q, Hayami T, Chen J, Wang W, Kapila S (2006). Relaxin and β-estradiol modulate targeted matrix degradation in specific synovial joint fibrocartilages: progesterone prevents matrix loss. Arthritis Res Ther..

[18] Yang JH, Kim JH, Lim DS, Oh KJ (2012). Effect of combined sex hormone replacement on bone/cartilage turnover in a murine model of osteoarthritis. Clin Orthop Surg..

[19] Funck-Brentano T, Lin H, Hay E, Ah Kioon MD, Schiltz C, Hannouche D (2012). Targeting bone alleviates osteoarthritis in osteopenic mice and modulates cartilage catabolism. PLoS One..

[20] Oestergaard S, Sondergaard BC, Hoegh-Andersen P, Henriksen K, Qvist P, Christiansen C (2006). Effects of ovariectomy and estrogen therapy on type II collagen degradation and structural integrity of articular cartilage in rats: implications of the time of initiation. Arthritis Rheum..

[21] Gao WL, Wu LS, Zi JH, Wu B, Li YZ, Song YC (2015). Measurement of serum estrogen and estrogen metabolites in pre- and postmenopausal women with osteoarthritis using high-performance liquid chromatography-electrospray ionization-tandem mass spectrometry. Braz J Med Biol Res..

[22] Afzal S, Khanam A (2011). Serum estrogen and interleukin-6 levels in postmenopausal female osteoarthritis patients. Pak J Pharm Sci..

[23] Sniekers YH, Weinans H, van Osch GJ, van Leeuwen JP (2010). Oestrogen is important for maintenance of cartilage and subchondral bone in a murine model of knee osteoarthritis. Arthritis Res Ther..

[24] Yoshida A, Morihara T, Matsuda K, Sakamoto H, Arai Y, Kida Y (2012). Immunohistochemical analysis of the effects of estrogen on intraarticular neurogenic inflammation in a rat anterior cruciate ligament transection model of osteoarthritis. Connect Tissue Res..

[25] Wang XD, Kou XX, Meng Z, Bi RY, Liu Y, Zhang JN (2013). Estrogen aggravates iodoacetate-induced temporomandibular joint osteoarthritis. J Dent Res..

[26] Maneix L, Servent A, Poree B, Ollitrault D, Branly T, Bigot N, et al. Up-regulation of type II collagen gene by 17β-estradiol in articular chondrocytes involves Sp1/3, Sox-9, and estrogen receptor alpha. J Mol Med (Berl). 2014;92:1179–200. 10.1007/s00109-014-1195-5 25081415

[27] Imgenberg J, Rolauffs B, Grodzinsky AJ, Schunke M, Kurz B (2013). Estrogen reduces mechanical injury-related cell death and proteoglycan degradation in mature articular cartilage independent of the presence of the superficial zone tissue. Osteoarthritis Cartilage..

[28] Kumagai K, Imai S, Toyoda F, Okumura N, Isoya E, Matsuura H (2012). 17β-Oestradiol inhibits doxorubicin-induced apoptosis via block of the volume-sensitive Cl(–) current in rabbit articular chondrocytes. Br J Pharmacol..

[29] Huang JG, Xia C, Zheng XP, Yi TT, Wang XY, Song G (2011). 17β-Estradiol promotes cell proliferation in rat osteoarthritis model chondrocytes via PI3K/Akt pathway. Cell Mol Biol Lett..

[30] Watt FE (2016). Hand osteoarthritis, menopause and menopausal hormone therapy. Maturitas..

[31] Nevitt MC, Cummings SR, Lane NE, Hochberg MC, Scott JC, Pressman AR (1996). Association of estrogen replacement therapy with the risk of osteoarthritis of the hip in elderly white women. Study of Osteoporotic Fractures Research Group. Arch Intern Med.

[32] Cirillo DJ, Wallace RB, Wu L, Yood RA (2006). Effect of hormone therapy on risk of hip and knee joint replacement in the Women’s Health Initiative. Arthritis Rheum..

[33] Carbone LD, Nevitt MC, Wildy K, Barrow KD, Harris F, Felson D (2004). The relationship of antiresorptive drug use to structural findings and symptoms of knee osteoarthritis. Arthritis Rheum..

[34] Seo SK, Yang HI, Lim KJ, Jeon YE, Choi YS, Cho S (2012). Changes in serum levels of cartilage oligomeric matrix protein after estrogen and alendronate therapy in postmenopausal women. Gynecol Obstet Invest..

[35] Spector TD, Nandra D, Hart DJ, Doyle DV (1997). Is hormone replacement therapy protective for hand and knee osteoarthritis in women?: The Chingford Study. Ann Rheum Dis..

[36] Hannan MT, Felson DT, Anderson JJ, Naimark A, Kannel WB (1990). Estrogen use and radiographic osteoarthritis of the knee in women. The Framingham Osteoarthritis Study. Arthritis Rheum..

[37] Zhang Y, McAlindon TE, Hannan MT, Chaisson CE, Klein R, Wilson PW (1998). Estrogen replacement therapy and worsening of radiographic knee osteoarthritis: the Framingham Study. Arthritis Rheum..

[38] Hart DJ, Doyle DV, Spector TD (1999). Incidence and risk factors for radiographic knee osteoarthritis in middle-aged women: the Chingford Study. Arthritis Rheum..

[39] Wise BL, Niu J, Zhang Y, Felson DT, Bradley LA, Segal N (2013). The association of parity with osteoarthritis and knee replacement in the multicenter osteoarthritis study. Osteoarthritis Cartilage..

[40] Liu B, Balkwill A, Cooper C, Roddam A, Brown A, Beral V (2009). Reproductive history, hormonal factors and the incidence of hip and knee replacement for osteoarthritis in middle-aged women. Ann Rheum Dis..

[41] Burr DB, Gallant MA (2012). Bone remodelling in osteoarthritis. Nat Rev Rheumatol..

[42] Pastoureau PC, Chomel AC, Bonnet J (1999). Evidence of early subchondral bone changes in the meniscectomized guinea pig. A densitometric study using dual-energy X-ray absorptiometry subregional analysis. Osteoarthritis Cartilage.

[43] Herrero-Beaumont G, Roman-Blas JA, Largo R, Berenbaum F, Castaneda S (2011). Bone mineral density and joint cartilage: four clinical settings of a complex relationship in osteoarthritis. Ann Rheum Dis..

[44] Komm BS, Mirkin S (2014). An overview of current and emerging SERMs. J Steroid Biochem Mol Biol..

[45] Tsai CL, Liu TK (1992). Inhibition of estradiol-induced early osteoarthritic changes by tamoxifen. Life Sci..

[46] Rosner IA, Boja BA, Goldberg VM, Moskowitz RW (1983). Tamoxifen therapy in experimental osteoarthritis. Curr Ther Res..

[47] Colombo C, Butler M, Hickman L, Selwyn M, Chart J, Steinetz B (1983). A new model of osteoarthritis in rabbits. II. Evaluation of anti-osteoarthritic effects of selected antirheumatic drugs administered systemically. Arthritis Rheum.

[48] Rosner IA, Malemud CJ, Goldberg VM, Papay RS, Getzy L, Moskowitz RW. Pathologic and metabolic responses of experimental osteoarthritis to estradiol and an estradiol antagonist. Clin Orthop Relat Res. 1982;171:280–6. 7140079

[49] Hoegh-Andersen P, Tanko LB, Andersen TL, Lundberg CV, Mo JA, Heegaard AM (2004). Ovariectomized rats as a model of postmenopausal osteoarthritis: validation and application. Arthritis Res Ther..

[50] Christgau S, Tanko LB, Cloos PA, Mouritzen U, Christiansen C, Delaisse JM (2004). Suppression of elevated cartilage turnover in postmenopausal women and in ovariectomized rats by estrogen and a selective estrogen-receptor modulator (SERM). Menopause..

[51] Andersson A, Bernardi AI, Stubelius A, Nurkkala-Karlsson M, Ohlsson C, Carlsten H, et al. Selective oestrogen receptor modulators lasofoxifene and bazedoxifene inhibit joint inflammation and osteoporosis in ovariectomised mice with collagen-induced arthritis. Rheumatology. 2015; kev355. 10.1093/rheumatology/kev355 PMC4746431 26424839

[52] Saito M, Kida Y, Nishizawa T, Arakawa S, Okabe H, Seki A (2015). Effects of 18-month treatment with bazedoxifene on enzymatic immature and mature cross-links and non-enzymatic advanced glycation end products, mineralization, and trabecular microarchitecture of vertebra in ovariectomized monkeys. Bone..

[53] Kavas A, Cagatay ST, Banerjee S, Keskin D, Tezcaner A (2013). Potential of Raloxifene in reversing osteoarthritis-like alterations in rat chondrocytes: an in vitro model study. J Biosci..

[54] Tinti L, Niccolini S, Lamboglia A, Pascarelli NA, Cervone R, Fioravanti A (2011). Raloxifene protects cultured human chondrocytes from IL-1β induced damage: a biochemical and morphological study. Eur J Pharmacol..

[55] Hooshmand S, do Soung Y, Lucas EA, Madihally SV, Levenson CW, Arjmandi BH (2007). Genistein reduces the production of proinflammatory molecules in human chondrocytes. J Nutr Biochem.

[56] Karsdal MA, Bay-Jensen AC, Henriksen K, Christiansen C (2012). The pathogenesis of osteoarthritis involves bone, cartilage and synovial inflammation: may estrogen be a magic bullet?. Menopause Int..

[57] Yoshii T, Yamada M, Minami T, Tsunoda T, Sasaki M, Kondo Y (2015). The effects of bazedoxifene on bone, glucose, and lipid metabolism in postmenopausal women with type 2 diabetes: an exploratory pilot study. J Clin Med Res..

[58] Palacios S, Silverman SL, de Villiers TJ, Levine AB, Goemaere S, Brown JP (2015). A 7-year randomized, placebo-controlled trial assessing the long-term efficacy and safety of bazedoxifene in postmenopausal women with osteoporosis: effects on bone density and fracture. Menopause..

[59] Beck TJ, Fuerst T, Gaither KW, Sutradhar S, Levine AB, Hines T (2015). The effects of bazedoxifene on bone structural strength evaluated by hip structure analysis. Bone..

[60] Pinkerton JV, Harvey JA, Lindsay R, Pan K, Chines AA, Mirkin S (2014). Effects of bazedoxifene/conjugated estrogens on the endometrium and bone: a randomized trial. J Clin Endocrinol Metab..

[61] Kim K, Svedbom A, Luo X, Sutradhar S, Kanis JA (2014). Comparative cost-effectiveness of bazedoxifene and raloxifene in the treatment of postmenopausal osteoporosis in Europe, using the FRAX algorithm. Osteoporos Int..

[62] Fujita T, Fujii Y, Munezane H, Ohue M, Takagi Y (2010). Analgesic effect of raloxifene on back and knee pain in postmenopausal women with osteoporosis and/or osteoarthritis. J Bone Miner Metab..

[63] Hattori Y, Kojima T, Kato D, Matsubara H, Takigawa M, Ishiguro N (2012). A selective estrogen receptor modulator inhibits tumor necrosis factor-alpha-induced apoptosis through the ERK1/2 signaling pathway in human chondrocytes. Biochem Biophys Res Commun..

[64] Funck-Brentano T, Cohen-Solal M (2015). Subchondral bone and osteoarthritis. Curr Opin Rheumatol..

[65] Bellido M, Lugo L, Roman-Blas JA, Castaneda S, Caeiro JR, Dapia S (2010). Subchondral bone microstructural damage by increased remodelling aggravates experimental osteoarthritis preceded by osteoporosis. Arthritis Res Ther..

[66] Liang Y, Duan L, Xiong J, Zhu W, Liu Q, Wang D (2016). E2 regulates MMP-13 via targeting miR-140 in IL-1β-induced extracellular matrix degradation in human chondrocytes. Arthritis Res Ther..

[67] Varady NH, Grodzinsky AJ (2016). Osteoarthritis year in review 2015: mechanics. Osteoarthr Cartil..

[68] Levillain A, Boulocher C, Kaderli S, Viguier E, Hannouche D, Hoc T (2015). Meniscal biomechanical alterations in an ACLT rabbit model of early osteoarthritis. Osteoarthritis Cartilage..

[69] Kato M, Takaishi H, Yoda M, Tohmonda T, Takito J, Fujita N (2010). GRIP1 enhances estrogen receptor alpha-dependent extracellular matrix gene expression in chondrogenic cells. Osteoarthritis Cartilage..

[70] Dai G, Li J, Liu X, Liu Q, Liu C (2005). The relationship of the expression of estrogen receptor in cartilage cell and osteoarthritis induced by bilateral ovariectomy in guinea pig. J Huazhong Univ Sci Technolog Med Sci..

[71] Li ZX, Chen CJ (2015). The effect of lowered serum levels of estrogen on the expression of ERα, ERβ, OPG and RANKL in rat mandibular condylar cartilage. Shanghai Kou Qiang Yi Xue..

[72] Song XX, Yu YJ, Li XF, Liu ZD, Yu BW, Guo Z (2014). Estrogen receptor expression in lumbar intervertebral disc of the elderly: gender- and degeneration degree-related variations. Joint Bone Spine..

[73] Komm BS, Mirkin S (2012). The tissue selective estrogen complex: a promising new menopausal therapy. Pharmaceuticals (Basel).

[74] Komm BS, Morgenstern D, Yamamoto AL, Jenkins SN (2015). The safety and tolerability profile of therapies for the prevention and treatment of osteoporosis in postmenopausal women. Expert Rev Clin Pharmacol..

[75] Lindsay R, Gallagher JC, Kagan R, Pickar JH, Constantine G (2009). Efficacy of tissue-selective estrogen complex of bazedoxifene/conjugated estrogens for osteoporosis prevention in at-risk postmenopausal women. Fertil Steril..

[76] Komm BS, Vlasseros F, Samadfam R, Chouinard L, Smith SY (2011). Skeletal effects of bazedoxifene paired with conjugated estrogens in ovariectomized rats. Bone..

